# Long-read PacBio genome sequencing of four environmental saprophytic *Sporothrix* species spanning the pathogenic clade

**DOI:** 10.1186/s12864-022-08736-w

**Published:** 2022-07-12

**Authors:** Weian Du, Domenico Giosa, Junkang Wei, Letterio Giuffrè, Ge Shi, Lamya El Aamri, Enrico D’Alessandro, Majida Hafidi, Sybren de Hoog, Orazio Romeo, Huaiqiu Huang

**Affiliations:** 1 Department of Dermatology and Venereology, the Third Affiliated Hospital of Sun Yat-sen University, Guangzhou, Guangdong China; 2 Department of Chemical, Biological, Pharmaceutical and Environmental Sciences, University of Messina, Messina, Italy; 3 School of Pharmaceutical Sciences, Sun Yat-sen University, Guangzhou, Guangdong China; 4 Medical Cosmetic and Plastic Surgery Center, The Sixth Affiliated Hospital of Sun Yat-sen University, Guangzhou, Guangdong China; 5 Department of Biology, Moulay Ismail University, Zitoune, Meknes, Morocco; 6 Department of Veterinary Sciences, University of Messina, Messina, Italy; 7 Center of Expertise in Mycology of Radboud University Medical Center / Canisius Wilhelmina Hospital, Nijmegen, The Netherlands

**Keywords:** *Sporothrix phasma*, *Sporothrix curviconia*, *Sporothrix protearum*, *Sporothrix variecibatus*, Sporotrichosis, SMRT PacBio sequencing, Long-read sequencing, De novo assembly, Comparative genomics

## Abstract

**Background:**

The genus *Sporothrix* belongs to the order *Ophiostomatales* and contains mainly saprobic soil and plant fungi, although pathogenic species capable of causing human infections are also present. The whole-genomes of disease-causing species have already been sequenced and annotated but no *comprehensive* genomic resources for environmental *Sporothrix* species are available, thus limiting our understanding of the evolutionary origin of virulence-related genes and pathogenicity.

**Result:**

The genome assembly of four environmental *Sporothrix* species resulted in genome size of ~ 30.9 Mbp in *Sporothrix phasma*, ~ 35 Mbp in *S. curviconia*, ~ 38.7 Mbp in *S. protearum*, and ~ 39 Mbp in *S. variecibatus*, with a variable gene content, ranging from 8142 (*S. phasma*) to 9502 (*S. variecibatus*). The analysis of mobile genetic elements showed significant differences in the content of transposable elements within the sequenced genomes, with the genome of *S. phasma* lacking several class I and class II transposons, compared to the other Sporothrix genomes investigated. Moreover, the comparative analysis of orthologous genes shared by clinical and environmental *Sporothrix* genomes revealed the presence of 3622 orthogroups shared by all species, whereas over 4200 genes were species-specific single-copy gene products. Carbohydrate-active enzyme analysis revealed a total of 2608 protein-coding genes containing single and/or multiple CAZy domains, resulting in no statistically significant differences among pathogenic and environmental species. Nevertheless, some families were not found in clinical species. Furthermore, for each sequenced *Sporothrix* species, the mitochondrial genomes was assembled in a single circular DNA molecule, ranging from 25,765 bp (*S. variecibatus*) to 58,395 bp (*S. phasma*).

**Conclusion:**

In this study, we present four annotated genome assemblies generated using PacBio SMRT sequencing data from four environmental species: *S. curviconia, S. phasma, S. protearum* and *S. variecibatus* with the aim to provide a starting point for future comparative genome evolution studies addressing species diversification, ecological/host adaptation and origin of pathogenic lineages within the genus *Sporothrix*.

**Supplementary Information:**

The online version contains supplementary material available at 10.1186/s12864-022-08736-w.

## Introduction

The genus *Sporothrix* belongs to the order *Ophiostomatales* and includes environmental fungi with a saprobic lifestyle in soil, plants, and decaying organic matter [1, 2]. These fungi are widely distributed across a variety of climates in the world and populate a wide range of natural habitats, in particular bark, infructescences of *Protea* plants and wood of different trees [1, 3, 4]. Some species are also pathogenic for humans and other animals and cause a cutaneous or extracutaneous type of infection known as sporotrichosis [5]. This infection is generally caused by only four (*Sporothrix schenckii*, *S. brasiliensis, S. globosa* and *S. luriei*) of the 62 species currently listed in the genus [4]. The disease affects mainly humans and felines, sometimes involving thousands of individuals in large epidemics and/or epizootics [6–8]. The pathogenic species constitute what is now commonly called “pathogenic clade” [1, 8] and some of them, especially *S. brasiliensis*, represent a serious emerging public health problem*.* This is highlighted by a recent report of the Pan American Health Organization/Regional Office of the World Health Organization (PAHO/WHO) which encouraged Latin American countries to raise awareness among doctors and veterinarians about the threat of *S. brasiliensis* in this particular geographical area [9, 10]. However, despite the growing incidence of sporotrichosis observed worldwide, genomic knowledge of pathogenic and/or environmental *Sporothrix* species is still very limited [11]. Little progress has been made in exploring the genetic changes implicated in genome evolution and species diversification, including ecological/host adaptation and origin of pathogenic lineages. This gap in knowledge reflects the lack of sequenced and annotated genomes for most *Sporothrix* species, in particular those of environmental origin; only the genomes of the major pathogenic species have as yet been sequenced and published [12–15].

A comparative analysis of *S. schenckii* and *S. brasiliensis* genomes revealed a remarkable variation in their transposon content as well as the exclusive presence of genes encoding homing endonucleases (HEs) in the large *S. brasiliensis* mitogenome [12]. HEs are highly specific DNA-cutting enzymes, widespread in all microbes including phage, mitochondria and chloroplasts [16], and can be classified into at least four families (GIY-YIG, LAGLIDADG, His-Cys box, and HNH), based on the presence of highly conserved characteristic amino acid motifs in the catalytic domain and active site of the protein [17]. These enzymes can be encoded by both free-standing genes or genes located within self-splicing elements such as group I, group II introns, and inteins [16, 17]. However, only genes coding GIY-YIG and LAGLIDADG HEs, have so far been found in fungal mitogenomes [17], including *S. brasiliensis* mtDNA [12]. No HEs have been detected in other previously sequenced *Sporothrix* genomes.

In this study, we decided to sequence, assembly and annotate the whole-genomes of four environmental saprobic *Sporothrix* species (*S. protearum, S. variecibatus, S. curviconia and S. phasma)* spanning the genus *Sporothrix* in order to provide genetic information on rapidly evolving genes, their functional importance and their role in host-pathogen interaction. The four species under study belong to distinct species complexes or form unique lineages in the genus *Sporothrix. Sporothrix protearum* was first collected from *Protea caffra* infructescences and is grouped in a subclade of the *S. stenoceras* complex containing only species collected from *Protea* plants [1]. *Sporothrix variecibatus* is also found in *Protea* spp., as well as in mites acting as vectors of fungal spores between these plants [18]. This species is part of the *S. gossypina* complex which is more distantly related to members of the pathogenic clade than *S. protearum*. Also *S. curviconia*, recovered from *Terminalia ivorensis* tree, is phylogenetically distant from pathogenic species, positioned within the S*porothrix* group G, close to the species *Sporothrix nebularis* and *Sporothrix nigrograna* [1]. Finally, *S. phasma*, first isolated from the infructescence of *Protea laurifolia* and *Protea neriifolia* by Roets et al. [19], forms a unique and exclusive genetic lineage (lineage E) which is sister to the pathogenic clade and represents, to date, the environmental, non-pathogenic, species phylogenetically closest to clinical taxa [1].

## Materials and methods

### Fungal strains and DNA extraction

The whole genomes of four environmental *Sporothrix* species (*S. curviconia* CBS 959.73, *S. phasma* CBS 119588, *S. protearum* CBS 116654 and *S. variecibatus* CBS 121960), obtained from the CBS*-*KNAW culture collection (Westerdijk Fungal Biodiversity Institute, The Netherlands), were sequenced in this study (Table 1).

**Table 1:** Genome statistics and gene content of nuclear and mitochondrial *Sporothrix* genomes examined in this study

Nuclear genome statistics	***S. phasma*** CBS 119588	***S. protearum*** CBS 116654	***S. variecibatus*** CBS 121960	***S. curviconia*** CBS 959.73
Total sequenced bases	883,093,002	947,624,674	919,509,964	542,611,844
Number of raw reads	128,870	109,238	106,098	87,407
Mean raw read length (bp)	6852.6	8674.9	8666.6	6207.9
Maximum raw read length (bp)	50,542	44,789	41,751	41,000
Number of corrected reads	114,302	104,897	101,452	78,181
Mean corrected read length (bp)	4889.5	6870.3	6897.2	4994.7
Maximum corrected read length (bp)	48,834	41,999	41,488	37,743
Mapped reads (%)	95.7	97.3	96.5	93.4
Number of total contigs	140	40	21	433
Largest contig (bp)	1,661,333	5,717,463	6,938,270	801,994
Genome size (bp)	30,907,658	38,728,587	38,959,714	35,054,974
GC content (%)	57.1	52.2	52.8	54.6
Coverage depth (mean)	23x	22x	22x	13x
Coverage ≥1x (%)	99.98	100	100	99.94
N50 (bp)	524,569	1,791,310	4,206,442	153,870
N75 (bp)	277,398	1,374,249	3,677,956	83,002
L50 (bp)	20	6	4	71
L75 (bp)	39	12	6	149
Total genes	8142	8691	9502	8519
Protein-coding genes	7916	8443	9289	8330
Ribosomal RNAs (rRNAs)	25	40	22	21
Transfer RNAs (tRNAs)	201	208	191	168
Pseudo-tRNAs	25	14	12	16
**Mitochondrial genome statistics**				
Number of total contigs	1	1	1	1
Mitogenome size (bp)	58,395	32,517	25,765	33,128
GC content (%)	24.8	24.9	25.7	24.8
Number of mapped reads	4700	903	922	1504
Coverage depth (mean)	414x	144x	144x	211x
Total genes	56	43	40	44
Protein-coding genes	25	16	15	18
Ribosomal RNAs (rRNAs)	2	2	2	2
Transfer RNAs (tRNAs)	26	25	23	24

Total genomic DNA was isolated using the FastDNA™ SPIN Kit (MP Biomedicals, China) following the manufacturer’s instructions. The integrity of the DNA molecules was checked using agarose gel electrophoresis and the purity of each sample was evaluated spectrophotometrically by measuring the absorbance A_260_/A_280_ and A_260_/A_230_ ratios. High-quality DNA (A_260/280_ ≥ 1.8) was used for library construction*.*

### Library preparation, genome sequencing and assembly

Fungal genomes were sequenced using the PacBio Sequel (SMRT) technology (Pacific Biosciences). Sequencing libraries were generated following PacBio’s protocol for the SMRTbell Template Prep. Kit 1.0-SPv3 (Pacific Biosciences). SMRTbell templates were annealed with the sequencing primer v3 and then bound to DNA polymerase 2.0 using the Sequel Binding Kit 2.0 according to the manufacturer’s recommendations (Pacific Biosciences). SMRTbell template DNA/polymerase complexes were captured and loaded onto PacBio Sequel SMRT Cell 1 M v2 using the MagBead Kit v2 (Pacific Biosciences). The SMRTbell libraries were sequenced using the Sequel Sequencing Kit 2.1 v2 chemistry.

After SMRT sequencing, for each *Sporothrix* genome, PacBio raw data were processed to obtain a high-quality *de-novo* genome assembly by using a combined bioinformatics approach based on the use of two different long-read assemblers, Canu v.2.0 [20] and wtdbg2 v.2.5 [21]. Raw reads were initially processed by Canu pipeline [20] which generates whole-genome assembly by operating in three distinct phases based on correction, trimming and assembling of the long-reads into uniquely-assemblable contigs, called unitigs [20]. Subsequently, raw reads were first corrected with CONSENT v.1.2.3 [22] and then used by wtdbg2 software [21] to produce a second *de-novo* genome assembly. The two draft genome assemblies were then merged using the C++ program *quickmerge* [23] to produce a more contiguous assembly which was subjected to a final refining process by using the “assembly polishing” function implemented in the CONSENT program [22]*.* Contigs that were less than 500 bp in length were removed from the assemblies and the QUAST program v.5.0.2 [24] was used to calculate assembly statistics and extract qualitative genomic metrics. Finally, the completeness of the genome assemblies was evaluated using BUSCO v*.*3.1.0 [25] by searching for conserved single-copy orthologs in the eukaryota_odb9, fungi_odb9 and ascomycota_odb9 lineage datasets [25]*.*

### Gene model prediction and functional annotation of *Sporothrix* genomes

*Sporothrix* genomes were annotated using the MAKER pipeline (v.3.00.0) [26] integrated with two ab-initio gene predictors, SNAP (v.2.39) [27] and AUGUSTUS (v. 3.3.1) [28], and two full data sets of proteins and expressed sequence tags (EST) sequences from *Ophiostomatales* (NCBI: txid5151) retrieved from the NCBI Protein and Nucleotide databases respectively (www.ncbi.nlm.nih.gov). Additional *S. schenckii* protein sequences were also downloaded from the “Sporothrix Genome DataBase” (http://sporothrixgenomedatabase.unime.it) [29] and included in the annotation analysis. However, before using these reference data sets in the annotation pipeline, we used the CD-HIT program v.4.8.1 [30] for clustering protein and/or EST sequences (similarity cut-off: 90%) in order to reduce the redundancy among them and obtain well-balanced data sets.

Functional annotations for the predicted gene models were performed using the PANNZER2 webserver [31].

The genome assemblies were also screened to detect repetitive and transposable elements (TEs) using the RepeatMasker v.4.1.0 (www.repeatmasker.org) software. Transfer RNAs (tRNAs) were predicted with the tRNAscan-SE software v.1.3.1 [32].

### Comparative genomics and phylogenomic analysis

Comparative analysis was performed using our four environmental *Sporothrix* genomes and previously sequenced and annotated genomes of members of the pathogenic (strains *S. schenckii* 1099–18, *S. brasiliensis* 5110 and *S. globosa* CBS 120340) and environmental strains (*S. pallida* SPA8 and *S. insectorum* RCEF 264 strains) clades of *Sporothrix* [12, 14, 15, 29, 33].

The program OrthoFinder v.2.3.11 [34] was used to perform protein orthology analysis by clustering sets of single or multi-copy orthologous genes across all *Sporothrix* species. An orthogroup was defined as a set of genes originating by speciation of a gene existing in the last common ancestor. For protein-based phylogenetic analysis, we selected only orthogroups containing singleton genes per species. *Neurospora crassa* OR74A (GenBank assembly accession: GCA_000182925.2) was also included and used as outgroup taxon according to previous studies [1]. The phylogenetic tree was generated by running the -M msa and –T raxml-ng commands in OrthoFinder [34] and the resulting tree visualized and edited by the web-based tool iTOL v3 [35]. Finally, to investigate the diversity of carbohydrate-active enzymes (CAZymes), encoded by *Sporothrix* genomes (CAZomes), we submitted the entire proteome of each species to the dbCAN2 meta server [36]. Only hits found in at least two databases were kept for comparative analysis of *Sporothrix* CAZomes. Each *Sporothrix* proteome was also submitted to KOfamKOALA web service [37] to retrieve all KEGG Orthology (KO) terms that were used by the KEGG Mapper tool [38] for linking KO annotation data to KEGG pathway maps and other biochemical frameworks.

The potential association between KO terms, and/or pathways, with pathogenic or environmental species was statistically evaluated using MaAsLin2 software [39].

### Assembly and annotation of mitochondrial *Sporothrix* genomes

**Fig. 1:**
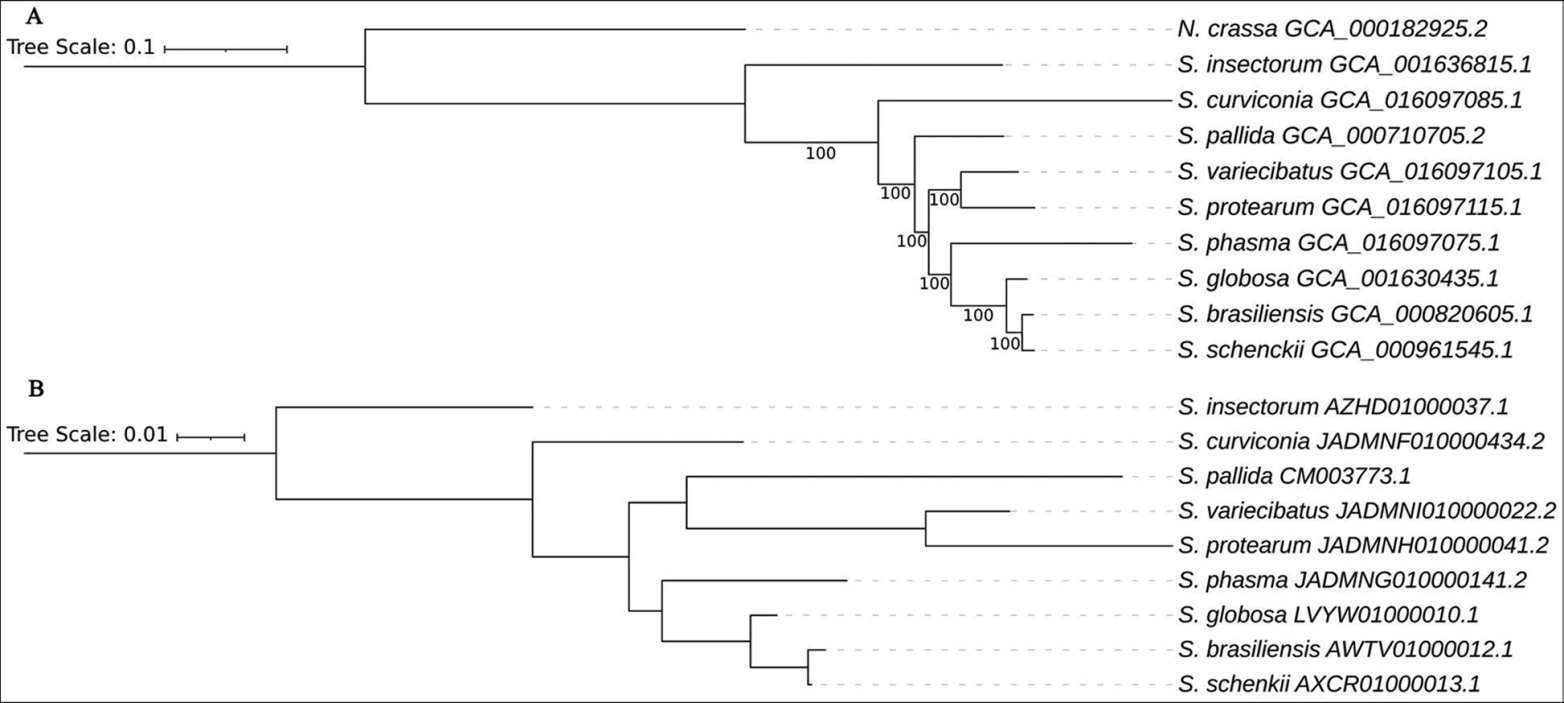
Nuclear (**A**) and mitochondrial (**B**) phylogenetic trees inferred by Maximum likelihood analysis of concatenated orthogroups containing single-copy representative proteins. The nuclear phylogeny is rooted on *N. crassa* and bootstrap values ≥98 are shown. *GenBank accession numbers of genome assemblies used in this study are listed after* species names

Mitochondrial genomes, including their corresponding mapping reads, were extracted from each *Sporothrix* genome assembly using the Samtools utility v.1.12 (www.htslib.org) and circlator bam2reads v.1.5.5 [40]. Reads were then assembled using the assemble and fixstart functions implemented in circlator [40]. The resulting mitochondrial genomes were finally annotated by Mitos2 [41] using the *S. schenckii* mitogenome as reference (GenBank accession n°: NC_015923.1) whereas tRNAs were predicted by ARWEN v1.2.3 [42] and tRNAscan-SE v2.0.7 [32]. Redundant features were manually removed from genome annotations. Additional whole-mitochondrial *Sporothrix* genomes, currently available in the GenBank database (Fig. 1), were downloaded and employed for comparative and phylogenetic analysis. Mitochondrial protein sequences were compared with OrthoFinder [34] and then used to generate a hierarchically clustered heatmap with the R package pheatmap v.1.0.12 (https://cran.r-project.org/web/packages/pheatmap/index.html).

Annotated homing endonucleases, LAGLIDADG (LD) and GIY-YIG (GIY) families, were classified by aligning our *Sporothrix* HE sequences with the whole fungal HE dataset reported in Megarioti and Koubelis [17]. A multiple alignment was generated by using the MAFFT program v. 7.453 [43] and then used as input for *phylogenetic* analysis inferred by FastTree 2 software v. 2.1.11 using the Whelan and Goldman (WAG) model of amino acid substitution [44].

## Results

### Characteristics of sequenced *Sporothrix* genomes

Overall statistics for nuclear and mitochondrial genome assemblies obtained in this study are shown in Table 1*.* The average read length of the PacBio corrected data set was > 6.8 Kbp for *S. protearum* and *S. variecibatus* and > 4.8 Kbp for *S. phasma* and *S. curviconia* respectively, with a maximum corrected read length ranging from 37,743 to 48,834 base pairs (Table 1).

PacBio data from *S. variecibatus* CBS 121960 generated the most complete and accurate genome assembly containing the lowest number of contigs (total contigs: 21; largest contig ~ 7 Mpb), compared to the other three assemblies (Table 1). The genome of this species was also the largest in size (38.9 Mbp) followed by that of *S. protearum* (38.7 Mbp), *S. curviconia* (35.0 Mbp), and *S. phasma* (30.9 Mbp) (Table 1). Except for *S. curviconia* (genome coverage: 13x) the average genome coverage depth for the other species was estimated to be ≥22x, while the genomic G + C contents were variable ranging from 52.2 in *S. protearum* to 57.1% in *S. phasma* (Table 1).

### Phylogenomics, gene content, and landscape of transposable elements

The number of nuclear genes predicted from each assembly was quite similar and ranged from 8142 in *S. phasma* to 9502 in *S. variecibatus*. *S. phasma* also showed the lowest number of protein-coding genes (7916) among the four sequenced *Sporothrix* genomes (Table 1). However, for most of the genome assemblies (*S. phasma*, *S. protearum* and *S. variecibatus*) we detected a high proportion of complete eukaryotic BUSCO genes (range: 79.5–95.7%; Supplementary Fig. S1) which confirms a high level of completeness of these genomes and a relatively low portion of fragmented or missing genes. Only *S. curviconia* showed slightly less complete BUSCO genes compared to other assemblies (Supplementary Fig. S1).

Prediction and analysis of mobile genetic elements revealed a significant difference in both type and abundance of TEs within sequenced genomes (Table 2). This marked difference was evident especially for class I TEs, or retrotransposons, which were particularly enriched in *S. protearum* genome (n° 664), followed by *S. variecibatus* (n° 434), *S. curviconia* (n° 363) and *S. phasma* (n° 140) (Table 2). The genome of this latter species was, in general, the least TEs-enriched with a total of 205 transposons detected, fewer than half of those found in *S. variecibatus* (n° 556) and *S. curviconia* (n° 481) genomes, and approximately one-quarter of those identified in *S. protearum* (n° 810) (Table 2). However, it is interesting to note that the *S. phasma* genome was found to be completely devoid of some class I (LTR TEs: ERVK, Ngaro and Pao; non-LTR TEs: LINEs I and R1) and class II (DNA TEs: CMC-EnSpm, Dada, Maverick, MULE-NOF, PIF-Harbinger, and Zisupton) transposons compared to all other *Sporothrix* genomes examined (Table 2). In particular, two LTR-transposons (Ngaro and Pao) were particularly abundant in non-*S. phasma* genomes (Table 2). By contrast, we identified two TEs (LINE-CR1 and MULE-MuDR) exclusively in the *S. phasma* genome which was also enriched by other TEs that were absent, or present in very low copy number, in the remaining genomes (Table 2). Finally, phylogenetic analysis based on orthologous protein sequences identified by OrthoFinder placed *S. phasma* as a sister lineage to the pathogenic clade, which contains only *Sporothrix* species of clinical interest (Fig. 1). On the other hand, *S. protearum* and *S. variecibatus* were grouped into two different but phylogenetically close related lineages, while *S. curviconia* was the environmental species most distant to the pathogenic clade (Fig. 1).

**Table 2:** Categories of transposable elements and simple and low complexity DNA repeats detected in *Sporothrix* genomes

Class I retrotransposons	***S. phasma*** CBS 119588	***S. protearum*** CBS 116654	***S. variecibatus*** CBS 121960	***S. curviconia*** CBS 959.73
Unidentified LTR element	0	4	2	0
LTR Copia	0	52	0	0
LTR DIRS	0	0	2	0
LTR ERV1	0	14	20	0
LTR ERVK	0	8	6	2
LTR ERVL	0	2	0	0
LTR ERVL-MaLR	0	0	0	2
LTR Gypsy	3	198	62	38
LTR Ngaro	0	126	104	98
LTR Pao	0	188	180	187
LINE CR1	39	0	0	0
LINE CR1-Zenon	0	2	0	0
LINE I	0	8	8	2
LINE I-Jockey	6	12	6	4
LINE L1	4	14	10	6
LINE L1-Tx1	0	4	0	4
LINE L2	25	2	2	0
LINE Penelope	9	4	2	0
LINE R1	0	4	2	4
LINE R2	0	0	0	2
LINE Rex-Babar	0	2	4	0
LINE RTE	0	0	0	2
LINE RTE-BovB	33	0	4	0
SINEs	21	20	20	12
**Total class I TEs**	**140**	**664**	**434**	**363**
**Class II DNA transposons**				
Unidentified DNA element	1	12	4	8
CMC-EnSpm	0	30	14	10
CMC-Transib	0	0	2	0
Crypton-A	0	2	0	0
Crypton-V	0	0	0	2
Dada	0	8	10	16
Ginger-1	0	2	2	0
hAT	0	2	0	2
hAT-Ac	34	42	38	26
hAT-Charlie	0	0	0	4
hAT-Tip100	0	2	0	2
Kolobok-T2	0	0	2	4
Maverick	0	4	2	2
Merlin	0	2	0	0
MULE-MuDR	1	0	0	0
MULE-NOF	0	2	10	6
PIF-Harbinger	0	4	4	2
TcMar	0	2	0	0
TcMar-ISRm11	0	0	0	2
TcMar-Tc1	0	0	4	2
TcMar-Tigger	7	0	0	0
Zisupton	0	6	4	12
RC_Helitron	22	26	26	18
**Total class II TEs**	**65**	**146**	**122**	**118**
**Total transposons detected**	**205**	**810**	**556**	**481**
**DNA repeats**				
Simple	15,733	145,298	125,360	163,539
Low complexity	1983	32,284	23,332	32,798

### Genome-wide identification of core and lineage-specific genes in *Sporothrix* spp.

A total of 13,485 orthogroups were found in our and previously sequenced *Sporothrix* genomes. Of these, 4271 (31.67%) were species-specific single-copy gene products distributed as follows: 1625 belonged to *S. schenckii*, 699 to *S. curviconia*, 660 to *S. insectorum*, 601 to *S. phasma*, 196 to *S. protearum*, 184 to *S. brasiliensis*, 147 to S. variecibatus, 116 to *S. pallida*, and 43 to *S. globosa*. Of the remaining 9214 orthogroups, 3622 (39.3%) defined the gene core shared by all species (2597 single-copy genes and 1025 multiple copies in at least one genome). Interestingly, 46 orthogroups (~ 0.3%), of which 29 containing hypothetical proteins, were shared exclusively by all members of the pathogenic clade while only 2, corresponding to “high-affinity iron permease ftrA” and “putative cyclase-domain-containing protein”, were found in environmental *Sporothrix* species (Supplementary Table S1).

**Fig. 2:**
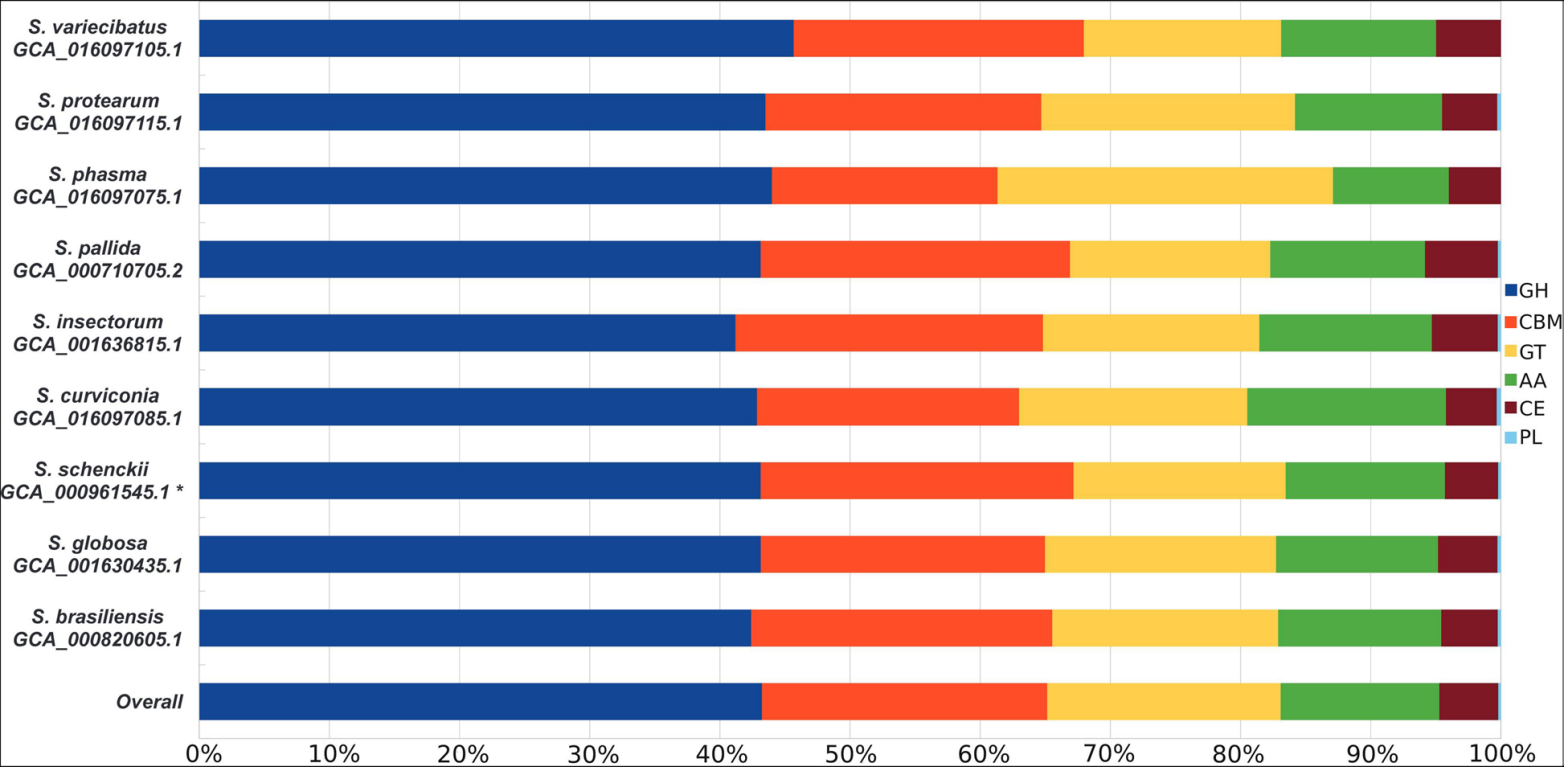
Barchart representation of CAZy families detected in *Sporothrix* genomes. Glycoside Hydrolases (GH), Glycosyl Transferases (GT), Carbohydrate-Binding Modules (CBM), Auxiliary Activities (AA), Carbohydrate Esterases (CE), Polysaccharide Lyases (PL). *The genome annotation (V2) used for the analysis was downloaded from the Sporothrix Genome DataBase

*Sporothrix* genome-wide analysis of carbohydrate-active enzyme diversity, identified a total of 2608 protein-coding genes containing single and/or multiple functional CAZy domains (total 3468) of which ~ 43.2% (n° 1499) were represented by glycoside hydrolases (GH), followed by carbohydrate binding modules (CBM) (n° 773; ~ 22.3%), glycosyl transferases (GT) (n° 604; ~ 17.4%), auxiliary activities (AA) (n° 426; ~ 12.3%), carbohydrate esterase (CE) (n° 159; ~ 4.6%), and polysaccharide lyases (PL) (n° 7; 0.2%) (Fig. 2). No statistically significant differences were observed among clinical and environmental species (*P* > 0.05 t-student test) in all classes of CAZymes. However, despite no CAZy families were specifically associated with clinical or environmental *Sporothrix* species, some families were missing in pathogenic (GH128, GH29, GH23, GH27, CBM23, CE2, PL26, GT109, GT31, and GT43), or environmental (CBM38 and CBM56) species, respectively (Supplementary Table S2). Moreover, among polysaccharide lyases, PL38 was found in both clinical and some environmental species (*S. insectorum* and *S. pallida*), whereas PL26 was detected only in S. curviconia and S. protearum. No PLs were detected in *S. phasma* and *S. variecibatus* genomes (Fig. 2; Supplementary Table S2).

Further functional characterization of *Sporothrix* genomes, identified a total 3484 unique KO terms of which 25 (~ 0.7%) and 111 (~ 3.2%) were exclusively detected in pathogenic and environmental species, respectively (Supplementary Table S3). Using KEGG Mapper Reconstruct Pathway tool, we reconstructed the complete maps of metabolic pathways for all *Sporothrix* species. This analysis revealed that pathways related to purine, aminoacid and glycan metabolism contained KEGG modules showing positive correlation (*p*-value ≤0.05) with pathogenic or environmental species (Supplementary Table S3). In particular, pathway modules involved in inosine monophosphate (KEGG module M00048) and ornithine biosynthesis (KEGG module M00028) were enriched in pathogenic species, while GABA shunt (KEGG module M00027) and N-glycosylation by oligosaccharyltransferase (KEGG module M00072) modules were most abundant in environmental species (Supplementary Table S3).

### *Sporothrix* mitogenomes and their variations

PacBio SMRT sequencing allowed to determine the complete sequence of all Sporothrix mitochondrial genomes which were assembled in a total of four single contigs consisting of circular DNA molecules of variable length ranging from 25,765 bp (*S. variecibatus*) to 58,395 bp (*S. phasma*) in length with an average GC content of ~ 25% (Table 1).

**Fig. 3:**
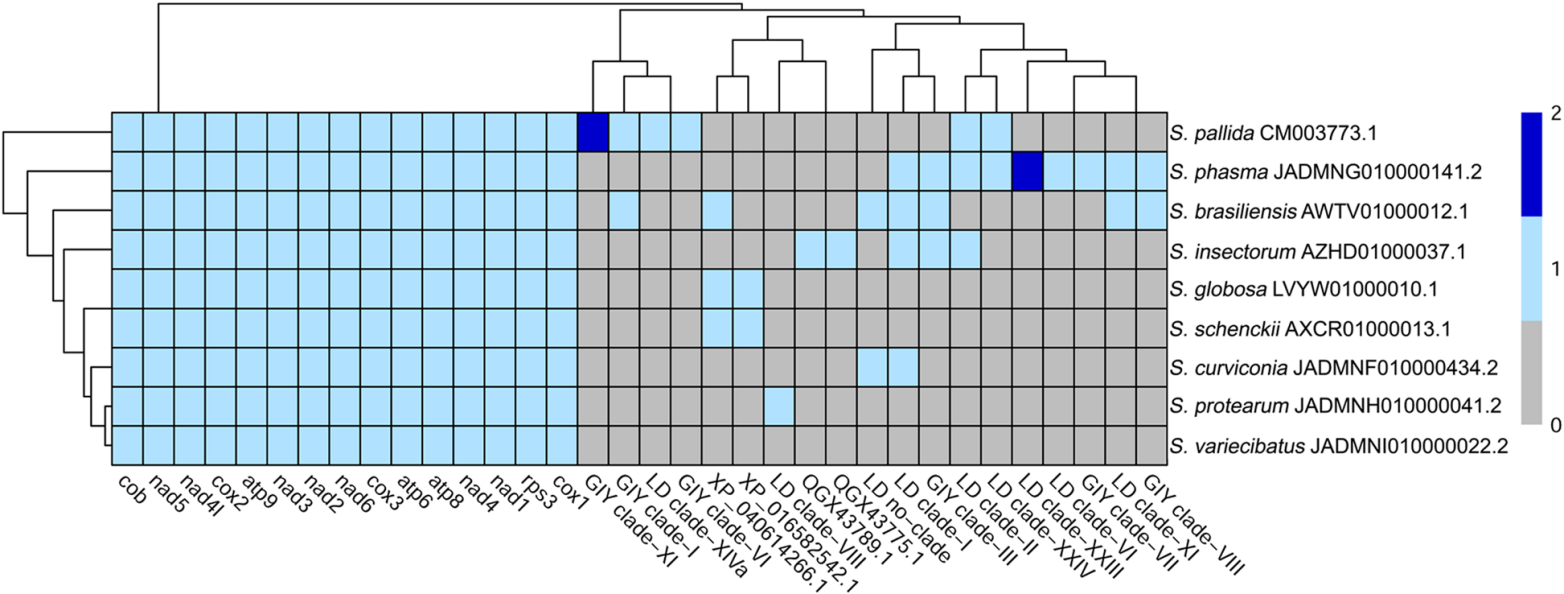
Heat-map showing both shared and taxon-specific protein-coding genes, including LDs and GIYs homing endonucleases, detected in mitochondrial genomes of clinical and environmental Sporothrix species included in this study. GenBank accession numbers of mitochondrial genome assemblies included in the comparative analysis are listed after species names

Although the total number of predicted genes varied slightly among the assemblies, we identified a distinctive 15-protein-coding core gene set shared by all *Sporothrix* mitogenomes investigated here (Fig. 3). However, additional ORFs (XP_040614266.1, XP_016582542.1, QGX43789.1 and QGX43775.1), encoding hypothetical proteins with unknown function, were predicted in both pathogenic and environmental species (Fig. 3). Interestingly, except for *S. variecibatus*, *S. globosa* and *S. schenckii*, the mitochondrial genomes of other *Sporothrix* species showed the presence of genes encoding for LD and GIY homing endonucleases (Fig. 3). More specifically, among the species sequenced in this study, *S. protearum* included one single clade-VIII LD (Fig. 3) located within the second intron of the *cox1* gene, while *S. curviconia* showed two diverse LDs, one located in the *nad3-*gene intron and not belonging to any clade (singleton LD), the other (clade-I LD) mapping in the *cox1*-gene intron 2, in fusion with the upstream exon 2. A large diversity of HEs was observed in the *S. phasma* mitogenome where 7 LDs and 3 GIYs from different clades were detected (Fig. 3). Among *S. phasma* LDs, 4 (clade-I, clade-II, clade-VI, and clade XI) were found in 3 of the 7 introns of the *cox1* gene; 2 clade-XXIII LDs were located in the first and last intron of the *nad2* gene respectively whereas one clade-XXIV LD was inserted in the *nad4* intron.

Regarding *S. phasma* GIY genes, one clade-VIII GIY was found in the atp6 intron while a clade-III GIY mapped in fusion with the upstream cox1-gene exon 6. A third GIY (clade-VII) was found as a free-standing ORF in the intergenic region between the proline tRNA (*trnP*) and *large subunit* ribosomal RNA (rnL) genes. However, despite the observed differences in HE content and other protein-coding genes (Fig. 3), *phylogenetic* relationships, inferred using the whole *mtDNA*-encoded *proteins,* were perfectly in agreement with current *Sporothrix* taxonomy defined by nuclear-gene phylogeny [1] (Fig. 1).

## Discussion

In the recent few years, sequencing of thousands of fungal genomes (http://jgi.doe.gov/fungi) has largely facilitated and stimulated many molecular studies concerning several aspects of the fundamental biology and physiology of fungi, including their phylogeny, evolution, and adaptation [45–47]. The first *Sporothrix* genome, that of *S. schenckii*, was sequenced and deposited in Genbank in 2013 (GenBank: GCA_000474925.1), and since then the genomes of all pathogenic species have been sequenced and released [12, 15] thereby providing useful background genetic information for future gene-specific analyses*.* Nevertheless, to date, no efforts have been made to sequence the genomes of related environmental species that differ in many phenotypic traits (i.e., virulence, pathogenicity, ecology, lifestyle, adaptation), thus preventing these traits to be explored in detail through comparison.

In this study, we provide genome sequences of four environmental *Sporothrix* species spanning the pathogenic clade within the genus *Sporothrix*. A preliminary comparison of genome size revealed variations in the environmental lineages with species showing either increasing (*S. protearum*, *S. variecibatus*, *S. curviconia*, *S. pallida*, *S. insectorum*) or decreasing (*S. phasma*) nuclear DNA content compared to members of the pathogenic clade. Interestingly, *S. phasma*, very close to the clinical species with which it shares close phylogenetic relationships [1], showed the smallest genome (~ 30.9 Mbp). In fact, previous phylogenetic studies, based on nuclear gene sequences, placed *S. phasma* nested with pathogenic species [1, 6] and we confirmed this close association using phylogenetic analysis of both nuclear and mitochondrial encoded proteins (Fig. 1).

Based on our bioinformatics data, we noticed that the reduction of the nuclear genome size in *S. phasma* correlates with the marked contraction of transposable elements observed in this species (Table 2). This unusual low-transposon density was also confirmed in a different *S. phasma* strain (CBS 119721; data not shown) which genome was recently sequenced using Illumina short-read sequencing data [48]. Moreover, it is also interesting to note that, parallel to the reduction of the nuclear genome, *S. phasma* CBS 119588 exhibits a remarkable expansion of its mitogenome (> 58 kbp; Table 1) which was also observed in the Illumina assembly of another strain of the same species (CBS 119721) [48]. This species possesses the largest mitochondrial genome among all *Sporothrix* species examined so far [12, 14, 15, 49]. In general, *Sporothrix* mitochondrial genomes show great diversity in size (Table 1) [12, 14, 15] which is a well-known phenomenon in fungi [17]. Comparative genome annotation revealed that both pathogenic and environmental *Sporothrix* species harbor 15 core protein-coding genes (*cob, cox1, cox2, cox3, atp6, atp8, atp9, nad1, nad2, nad3, nad4, nad4L, nad5, nad6,* and *rps3*) *(*Fig. 3*)* usually found in fungal mitogenomes [50]. However, except for *S. variecibatus*, additional ORFs, encoding hypothetical proteins or HEs, were also found (Fig. 3). In particular, HEs detected in this study belonged to LD and GIY families which were initially identified only in *S. brasiliensis*, but not in the *S. schenckii* [12] or *S. globosa* mitogenomes ([15]; Fig. 3). On the other hand, compared to *S. brasiliensis*, *S. phasma* harbours more HEs which are responsible for the mitogenome size expansion observed in this species. A similar trend was also observed in *S. pallida* which hosts 4 GIYs and 3 LDs (Fig. 3) but, unlike *S. phasma*, only one HE (GIY clade-I) was shared with *S. brasiliensis*. However, in general, *Sporothrix* HEs were highly diversified and classified in several distinct clades according to the recent phylogeny by Megarioti and Kouvelis [17]. Clade-I/Clade-II LDs and Clade-III GIYs appear to be the most common homing endonucleases in *Sporothrix* spp., but further studies, using more isolates, are needed to validate these data and confirm the absence of HE elements in *S. globosa* and *S. schenckii* populations.

Another important aspect of our work concerns the genome-wide analysis of metabolic pathways and genes encoding carbohydrate-active enzymes, which allowed us to detect the enrichment of specific metabolic KEGG modules and CAZy families in both pathogenic and environmental *Sporothrix* species (Supplementary Table S2 and Table S3). Most evident differences were observed in pathways related to purine, aminoacid and glycan metabolism. Interestingly, the KEGG pathway module M00048, involved in de novo synthesys of purine nucleotides, was enriched in clinical species, which is in agreement with previous studies reporting that this biosynthetic pathway is essential during host infection and that deletion of key enzymes in several fungal pathogens resulted in a reduced virulence and pathogenicity of the strains [51]. Moreover, several of the CAZy families found, such as CBM50, CBM18 and GH18, were previously reported to be markedly expanded in pathogenic *Sporothrix species* when compared to other *Sordariomycetes* and/or thermo-dimorphic fungal pathogens [12, 52]. Teixeira et al. [12], also observed a lack of polysaccharide lyase genes (CAZy PL family) in the *Sporothrix* lineage which was also confirmed in a recent comparative genomics study [52]. The lack of PL genes in *Sporothrix* spp. was interpreted as an evolutionary adaptation from saprobic/phytopathogenic to animal pathogenic lifestyle [12] *but,* unlike previous studies [12, 52], we detected a novel polysaccharide lyase family (PL38) [53] in clinical and some environmental species suggesting a continuous gene screening as well as a more in-depth comparative analysis of existing and future *Sporothrix* genomes. Caution should be taken when comparing some *Sporothrix* genome assemblies currently available in Genbank as they could be derived from still undescribed species or species whose description and/or naming needs to be re-evaluated [54]. This is the case in *S. insectorum* strain RCEF 264, which genetically deviates from the type strain of *S. insectorum* CBS 756.73 suggesting that a revision of its current taxonomic status is required [54].

In conclusion, the release of genome-wide sequence data of additional *Sporothrix* species is certainly a significant milestone for *Sporothrix* community because it sets the groundwork for future genetic studies and comparative genome analysis among *pathogenic and saprophytic* members of the *Sporothrix* lineage which evolved different lifestyles and host specificities.

## Supplementary Information

**Additional file 1: Supplementary Fig. S1.** Number of complete, fragmented, and missing orthologs obtained by BUSCO analysis for the four *Sporothrix* genomes sequenced in this study.

**Additional file 2.**

**Additional file 3.**

**Additional file 4.**

## Data Availability

The draft whole-genome sequences of the four *Sporothrix* species have been deposited at DDBJ/ENA/GenBank under the following accession numbers: JADMNF000000000 (*S. curviconia* CBS959.73), JADMNG000000000 (*S. phasma* CBS 119588), JADMNH000000000 (*S. protearum* CBS 116654) and JADMNI000000000 (*S. variecibatus* CBS 121960). PacBio SMRT raw reads have also been submitted into the Sequence Read Archive (SRA) database under the following accession numbers*:* SRX8367671-SRX8367674, associated with BioProject ID: PRJNA633855. The datasets generated and analysed during the current study are available in the SRA repository, https://www.ncbi.nlm.nih.gov/bi-oproject/?term=prjna633855.
