# Supplementary figures and images for: Long-read PacBio genome sequencing of four environmental saprophytic Sporothrix species spanning the pathogenic clade
Source: BMC Genomics. 2022 Jul 12;23:506. doi: 10.1186/s12864-022-08736-w (PMC9281073; doi:10.1186/s12864-022-08736-w)

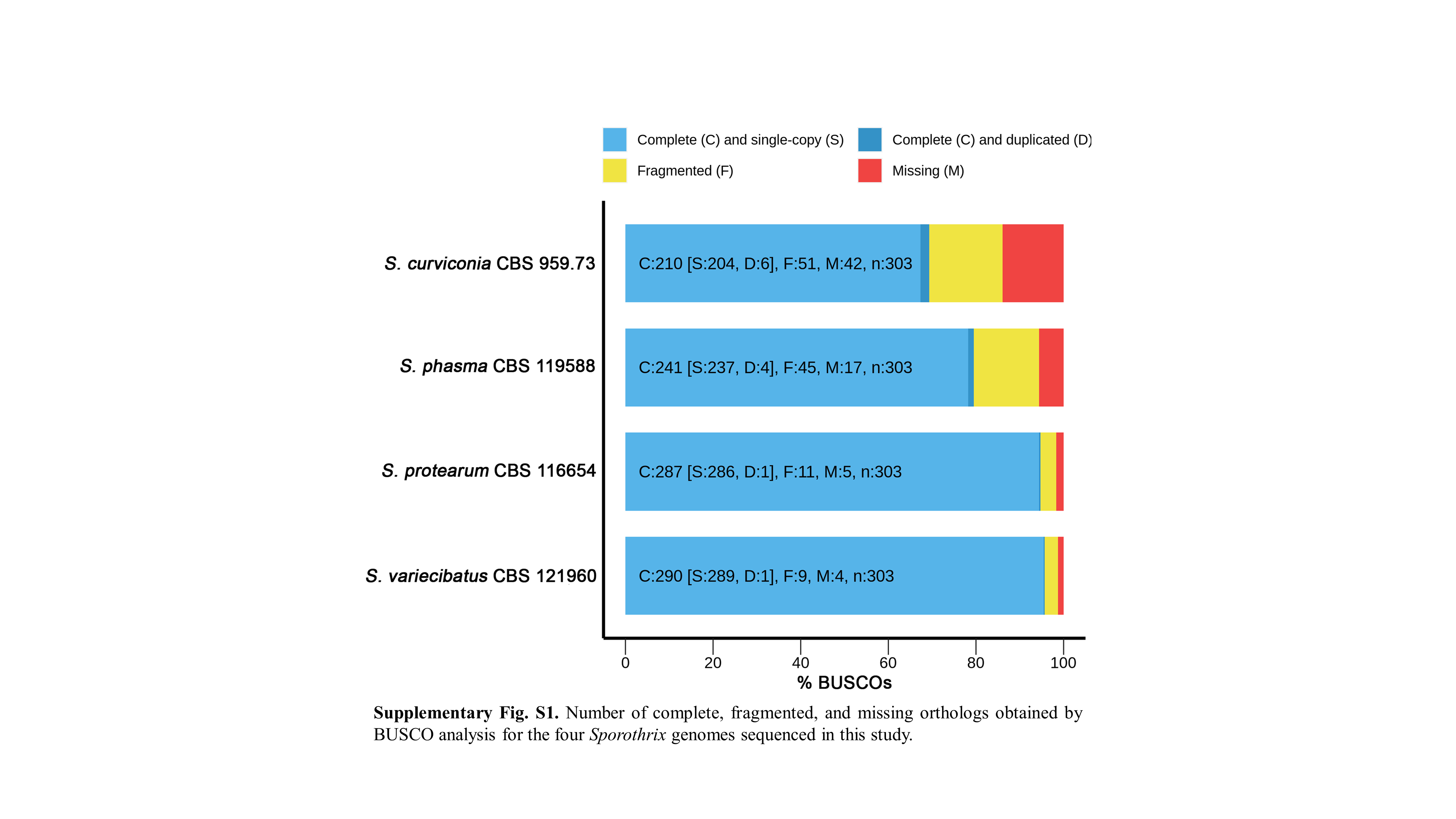

Supplement: Supplementary file 1 — Additional file 1: Supplementary Fig. S1. Number of complete, fragmented, and missing orthologs obtained by BUSCO analysis for the four Sporothrix genomes sequenced in this study. [file 12864_2022_8736_MOESM1_ESM.tif]
